# *Tbx1 *and *Brn4 *regulate retinoic acid metabolic genes during cochlear morphogenesis

**DOI:** 10.1186/1471-213X-9-31

**Published:** 2009-05-29

**Authors:** Evan M Braunstein, Dennis C Monks, Vimla S Aggarwal, Jelena S Arnold, Bernice E Morrow

**Affiliations:** 1 Department of Genetics, Albert Einstein College of Medicine, 1300 Morris Park Avenue, Bronx, New York, 10461, USA

## Abstract

**Background:**

In vertebrates, the inner ear is comprised of the cochlea and vestibular system, which develop from the otic vesicle. This process is regulated via inductive interactions from surrounding tissues. *Tbx1*, the gene responsible for velo-cardio-facial syndrome/DiGeorge syndrome in humans, is required for ear development in mice. *Tbx1 *is expressed in the otic epithelium and adjacent periotic mesenchyme (POM), and both of these domains are required for inner ear formation. To study the function of *Tbx1 *in the POM, we have conditionally inactivated *Tbx1 *in the mesoderm while keeping expression in the otic vesicle intact.

**Results:**

Conditional mutants (*TCre-KO*) displayed malformed inner ears, including a hypoplastic otic vesicle and a severely shortened cochlear duct, indicating that *Tbx1 *expression in the POM is necessary for proper inner ear formation. Expression of the mesenchyme marker *Brn4 *was also lost in the *TCre-KO*. *Brn4*^-^;*Tbx1*^+/-^embryos displayed defects in growth of the distal cochlea. To identify a potential signal from the POM to the otic epithelium, expression of retinoic acid (RA) catabolizing genes was examined in both mutants. *Cyp26a1 *expression was altered in the *TCre-KO*, while *Cyp26c1 *showed reduced expression in both *TCre-KO *and *Brn4*^-^;*Tbx1*^+/- ^embryos.

**Conclusion:**

These results indicate that *Tbx1 *expression in the POM regulates cochlear outgrowth potentially via control of local retinoic acid activity.

## Background

The vertebrate inner ear develops from the otic vesicle, which forms via invagination of specified ectodermal cells adjacent to the hindbrain. A crucial phase in development of the sensory structures of the inner ear occurs as the otic epithelium acquires regional identity along its axes. Classic explant experiments by developmental biologists first suggested that otic patterning occurred in response to signals from surrounding tissues, and recent work has revealed much of the molecular basis of these interactions [for review see [1-3]]. For example, genetic studies in the mouse showed that genes expressed along the dorsoventral axis of the otic vesicle are induced by signals from the roof plate and floor plate of the neural tube [4,5]. Specifically, members of the Wnt family of morphogens, secreted from the dorsal hindbrain, regulate the expression of dorsal genes required for development of vestibular structures, while Sonic hedgehog (Shh) secreted from the ventral hindbrain and notochord is necessary for the expression of ventral genes that promote cochlear formation.

Proper patterning of the otic vesicle is essential for subsequent growth and morphogenesis of the inner ear. In the ensuing stages of development, genes expressed within the otic vesicle are known to direct formation of the cochlea and vestibular structures. However, surrounding tissues continue to play an important role. *Shh *promotes outgrowth of the cochlear duct via activation of *Gli2 *and *Gli3 *in the otic vesicle and surrounding mesenchyme [6]. In addition, periotic mesenchyme (POM) cells condense around the otic vesicle and undergo chondrogenesis to give rise to the otic capsule, which surrounds and protects the inner ear sensory structures. The close association between the POM and the otic epithelium suggests that epithelial-mesenchymal interactions exist between these two tissues, and disruption of these interactions could lead to defects in inner ear formation. Indeed, multiple mouse mutants indicate that signaling from the epithelium to the mesenchyme is crucial for capsule formation and is dependent upon members of the *Fibroblast growth factor (Fgf) *and *Bone morphogenic protein (Bmp) *gene families [7-9].

Evidence supporting a requirement for signals from the POM to the otic vesicle during inner ear formation also exists. Explant experiments demonstrated that cultured otic vesicle epithelium failed to form differentiated hair cells in the absence of surrounding POM cells [10,11]. Furthermore, mice null for *Brn4*, a Pou-domain transcription factor expressed in the POM but not the otic vesicle, display a reduction in the number of turns of the cochlea [12]. These data indicate a cell nonautonomous role for the POM during inner ear development.

*TBX1 *is a member of a large family of transcription factors called T-box genes that have important roles in embryonic development. Haploinsufficiency of *TBX1 *in humans is associated with velo-cardio-facial syndrome/DiGeorge syndrome (VCFS/DGS), which causes sensorineural hearing loss in approximately 10% of patients [13]. In the mouse, *Tbx1 *is essential for the development of the inner ear, evidenced by the lack of both auditory and vestibular structures as well as an expansion of the cochleovestibular ganglion (CVG) in *Tbx1 *null mutants [14,15]. During embryogenesis, *Tbx1 *is expressed in both in the otic vesicle and in the ventral POM cells that lie between the source of *Shh *and the otic vesicle. Conditional ablation of *Tbx1 *expression in the otic vesicle results in an inner ear phenotype identical to that of *Tbx1*^-/- ^embryos, indicating a tissue autonomous role for *Tbx1 *in otic vesicle patterning [16]. However, the POM domain of *Tbx1 *was also shown to be essential for normal outgrowth of the cochlear duct, supporting a role for the mesenchyme in morphogenesis of the inner ear epithelium [17].

These data support a model in which cochlear outgrowth is regulated by Shh signaling from the ventral midline via genes expressed in the POM and the otic vesicle. Expression of both *Tbx1 *and *Brn4 *in the POM is responsive to Shh signaling, and is lost in *Shh *null mutants [4]. In contrast, *Tbx1 *expression in the otic vesicle is unaffected in *Shh*^-/- ^embryos [4,18]. This indicates that the mechanism by which *Shh *regulates cochlear duct outgrowth may be distinct from its regulation of otic vesicle patterning.

To further characterize the role of the POM in inner ear morphogenesis and identify genes downstream of *Tbx1 *and *Brn4 *signaling to the otic vesicle, we generated mutants in which *Tbx1 *was specifically ablated in the mesoderm. *T-Cre *mediated conditional mutants (*TCre-KO*) displayed severe defects in growth and coiling of the cochlear duct, similar to a comparable *Tbx1 *mutant using an alternative *Cre *driver [17]. To determine the molecular basis for these defects, Brn4 protein expression was examined in *TCre-KO *mutants, since adult *Brn4*^-^;*Tbx1*^+/- ^mice also display a reduction in the number of cochlear turns [19]. Expression of Brn4 was lost in the POM in conditional mutants, and *Brn4*^-^;*Tbx1*^+/- ^embryos exhibited similar but less severe defects in cochlear coiling compared to *TCre-KO *embryos. Further, expression of retinoic acid (RA) metabolizing enzymes was examined because RA has been implicated in the control of epithelial-mesenchymal interactions [20]. We identified changes in the expression of RA catabolizing enzymes *Cyp26a1 *and *Cyp26c1 *in both *TCre-KO *and *Brn4*^-^;*Tbx1*^+/-^mutants. These results support a role for *Tbx1 *and *Brn4 *in regulating levels of RA in the POM required for inner ear morphogenesis.

## Results

### The inner ears of *TCre-KO *embryos are malformed

To assess the role of *Tbx1 *in the POM, conditional mutants were created in which *Tbx1 *expression was ablated in all mesodermal tissues. This mutant was generated by crossing mice carrying the *Tbx1*^*flox *^allele with *T-Cre *transgenic mice to obtain *T-Cre;Tbx1*^*flox*/- ^embryos (*TCre-KO*). To confirm that Cre activity was restricted to the mesoderm, *T-Cre *mice were crossed with the *ROSA26 *reporter strain [21] and offspring were analyzed for *LacZ *expression. At E9.0 and E10.5, Cre activity was detected in the mesodermal cells surrounding the otic vesicle and not in the otic epithelium (Additional file 1, Fig. S1A, B). Analysis of *Tbx1 *expression at E9.5 and E10.5 in *TCre-KO *embryos revealed reduced *Tbx1 *expression in the mesoderm compared to control (*Tbx1*^+/-^) embryos, while *Tbx1 *expression in the otic vesicle was unaffected (Additional file 1, Fig. S1C-F). Analysis of *TCre-KO *embryos using an anti-Tbx1 antibody (Ab) confirmed that the residual *Tbx1 *expression was not due to an aberrant transcript (data not shown). These data indicated that the *TCre-KO *mutant was a mesodermal hypomorph. Supporting this, *TCre-KO *mutants displayed a phenotype that closely matched another *Tbx1 *mesodermal conditional mutant generated using the *Mesp1-Cre *driver [22]. Analysis of the inner ear of both mutants by paint-fill (n = 6) revealed slightly variable but overlapping phenotypes, however the most severely affected inners ear of *TCre-KO *embryos displayed a shorter cochlear duct than *Mesp1Cre-KO *mutants (Additional file 2, Fig. S2A-D). *Mesp1Cre-KO *mutants were also found to be hypomorphic following examination of *Tbx1 *expression (Additional file 2, Fig. S2E-F). Thus, mesodermal expression of *Tbx1 *in the *TCre-KO *mutant appears to be reduced to a level sufficient to isolate the role of *Tbx1 *in the POM.

**Figure 1:**
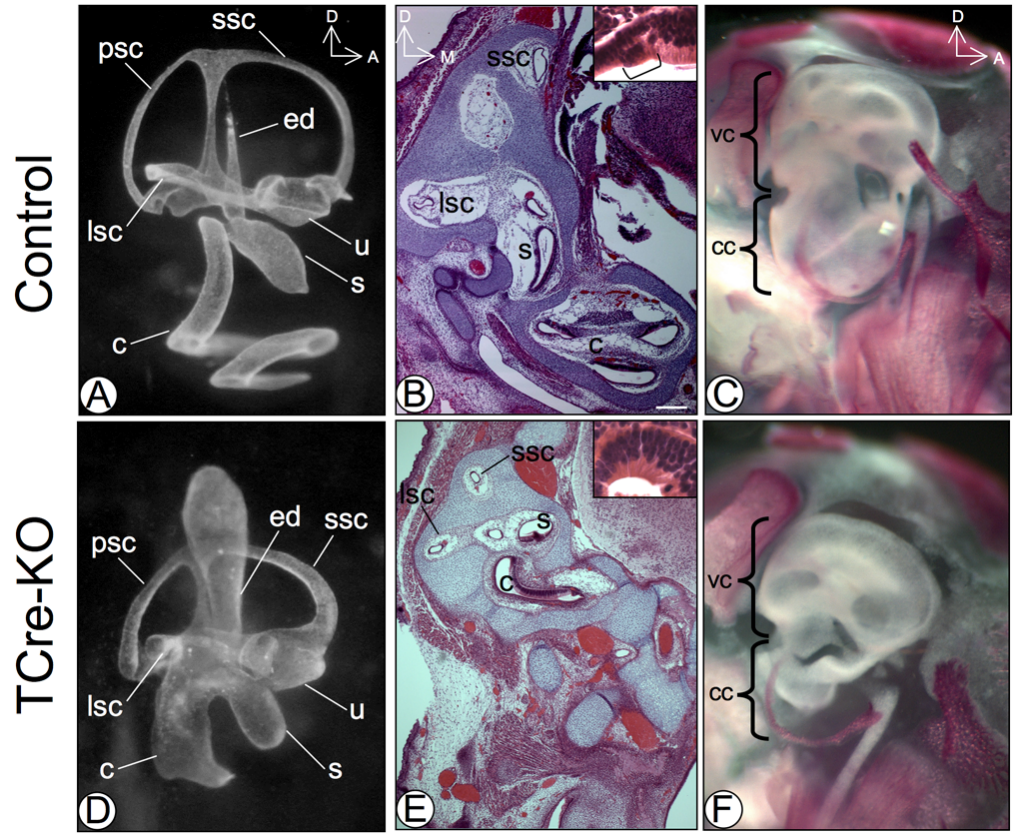
**Inner ear defects in the *TCre-KO***. At E15.5, a paint-filled inner ear of a control embryo (A) displays a cochlea (c) consisting of 1.75 turns and a vestibular system consisting of the saccule (s), utricle (u), endolymphatic duct (ed) and lateral, posterior and superior semicircular canals (lsc, psc, ssc). A transverse section though the inner ear of an E17.5 control embryo (B) displays the sensory structures of the ear with an intact organ of Corti (bracketed in inset) and the surrounding capsule. Bone and cartilage staining of an E17.5 control embryo (C) reveals the vestibular capsule (vc) and cochlear capsule (cc). Paint-fill of the inner ear of an E15.5 *TCre-KO *embryo (D) reveals malformations of both the auditory and vestibular system. The cochlea is severely shortened, the vestibular structures are hypoplastic and the endolymphatic duct (which did not completely fill with paint in (A)) is enlarged. At E17.5, the inner ears of *TCre-KO *embryos (E) are malformed and display only the hook region of the cochlea (c) with no recognizable organ of Corti (inset), and hypoplastic semicircular canals (lsc, ssc). Bone and cartilage staining (F) reveals a malformed cochlear capsule (cc), while the vestibular capsule (vc) appears relatively unaffected. Scale bar in B is 200 μm.

Examination of the membranous labyrinth of *TCre-KO *embryos revealed multiple defects. The cochlea normally forms one and three quarter turns in the mouse by E15.5 (Fig. 1A). At this stage, 4 of 6 *TCre-KO *embryos displayed a severely shortened cochlea with only the most basal, or hook region, present (Fig. 1D, Additional file 2 – Fig. S2B). Two *TCre-KO *embryos displayed an abnormal but slightly longer cochlea (Additional file 2, Fig. S2A). The vestibular system exhibited consistent mild hypoplasia, however the semicircular canals, utricle and saccule were always present. In addition, the endolymphatic duct and sac were enlarged (Fig. 1D), reminiscent of Mondini dysplasia. Histological sections through the inner ear of *TCre-KO *embryos at E17.5 (n = 8) confirmed the cochlear defects seen by paint-fill and revealed a lack of a recognizable organ of Corti (Fig. 1B and 1E). This is consistent with the sensory organ defects reported in *Mesp1Cre-KO *mutants, which displayed a lack of hair cells in the medial and apical cochlea evidenced by the absence of Myosin 7a expression [17]. In addition, *TCre-KO *embryos displayed abnormalities in the structure of the otic capsule (Fig. 1B, E). Interestingly, the capsule surrounding the vestibular system appeared relatively unaffected, while the cochlear capsule was severely malformed. This was easily appreciated upon bone and cartilage staining of *TCre-KO *embryos (n = 5; Fig. 1C, F). The capsule malformations may be due to defects in the POM, or secondary to the malformations of the membranous labyrinth.

### Otic patterning appears unaffected in *TCre-KO *mutants

**Figure 2:**
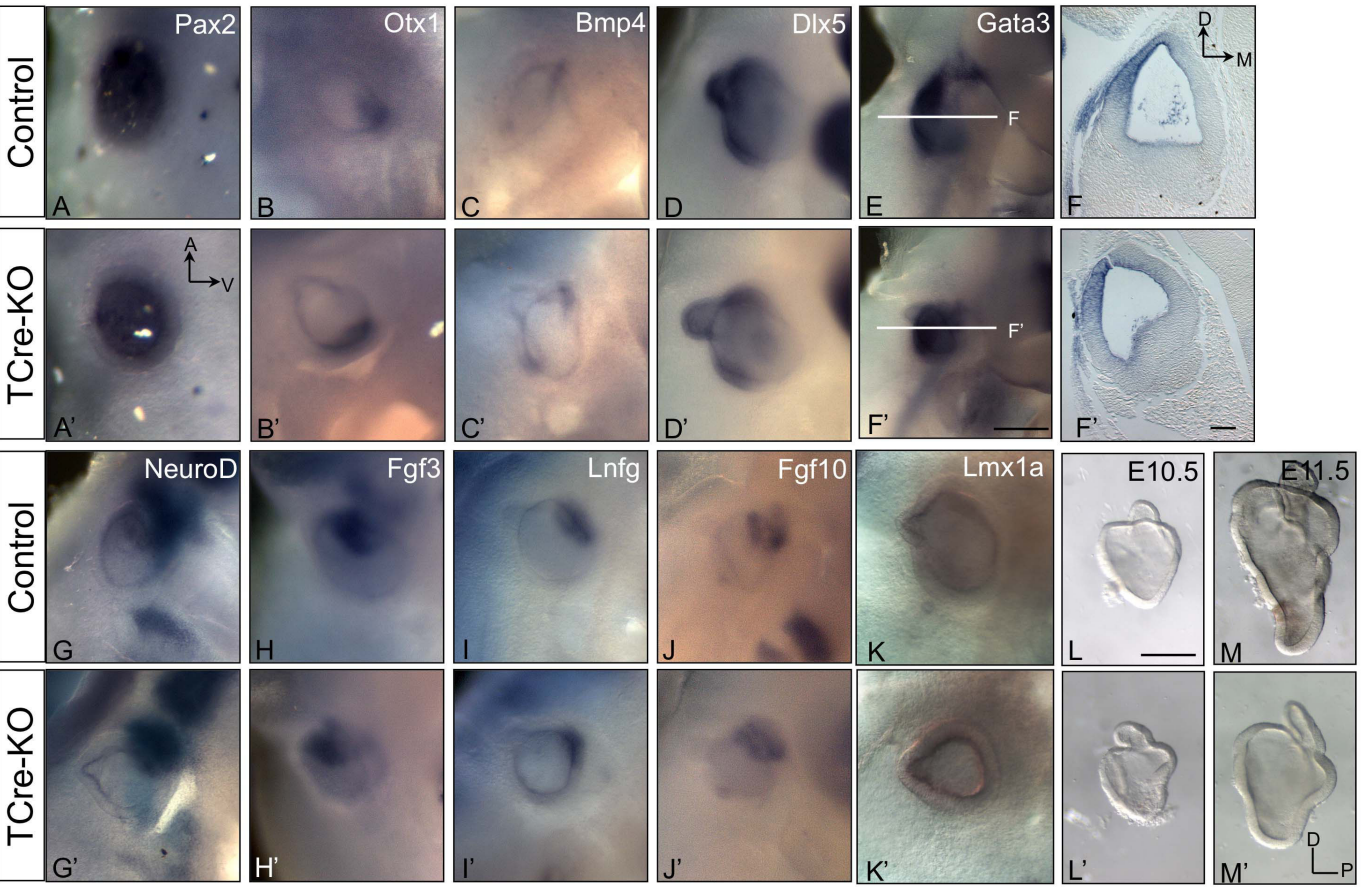
**Otic markers and vesicle morphology are unaffected at E10.5 in *TCre-KO *mutants**. Expression of sensory (A-F) and neurogenic (G-J) markers are unchanged in *TCre-KO *embryos compared to controls at E10.5. Ventral markers *Pax2 *and *Otx1*, as well as dorsal markers *Bmp4 *and *Dlx5 *are normal in *TCre-KO *mutants (A'-D'). *Gata3 *expression in the lateral otic vesicle is also unchanged in the *TCre-KO*, visualized in cross sections through the otic vesicle (F'). Expression of anterior markers *NeuroD*, *Fgf3*, *Lnfg *and *Fgf10 *display normal expression in the CVG in both mutants (G'-J') and controls (G-J). Expression of *Lmx1a*, which marks the growing endolymphatic duct, is also unchanged (K, K'). Microdissected otic vesicles are shown at E10.5 (L, L') and E11.5 (M, M'). At E10.5, the morphology of the otic vesicle in control (L) and *TCre-KO *(L') embryos is similar. By E11.5, the otic vesicles of *TCre-KO *embryos (M') are malformed and hypoplastic compared to controls (M). Scale bar in L is 250 μm and applies to all panels except (E, E') and (F, F'). Scale bars in E' and F' are 200 μm.

Ventral structures of the inner ear in *TCre-KO *embryos were more severely affected than dorsal structures, consistent with the localization of *Tbx1 *expression in the ventral POM during inner ear morphogenesis (Additional file 1, Fig. S1C). Because *Shh *is known to regulate the expression of ventral markers in the otic vesicle, we speculated that patterning of the dorsoventral axis might be abnormal in *TCre-KO *embryos. Analysis of ventral sensory markers *Pax2 *and *Otx1 *and dorsal markers *Bmp4 *and *Dlx5 *revealed no changes in expression in the otic vesicle of *TCre-KO *embryos versus controls at E10.5 (n = 3, Fig. 2A–D). This suggests that early dorsoventral patterning occurs normally in the absence of mesodermal *Tbx1*. In addition, expression of *Gata3*, a lateral marker, as well as anterior markers *NeuroD*, *Fgf10*, *Fgf3 *and *Lnfg*, was unchanged in *TCre-KO *mutants compared to controls at E10.5 (n = 3, Fig. 2E–J). *Lmx1a*, which has been shown to be required for development of the endolymphatic duct [23], is also unchanged in *TCre-KO *embryos at this stage (n = 3, Fig. 2K, K'). This indicates that early otic patterning and formation of the CVG are likely unaffected in *TCre-KO *mutants. This is consistent with the reported normal expression of neurogenic markers *Isl1 *and *Ngn1 *in *Mesp1Cre-KO *embryos at the same stage [24].

To ascertain the stage in which changes in gene expression might first appear in the *TCre-KO *mutants, we identified the onset of morphological defects in mutant embryos. At E10.5, the otic vesicles of *TCre-KO *embryos were indistinguishable in overall size and shape from control otic vesicles (n = 4; Fig. 2L, L'), consistent with the lack of expression changes of molecular markers. However, at E11.5 *TCre-KO *embryos exhibited a malformed and hypoplastic otic vesicle that was easily distinguished from controls (n = 9; Fig. 2M, M'). Thus, physical defects in the otic vesicles *TCre-KO *embryos do not appear until after axis determination is complete, consistent with a role for *Tbx1 *expression in the POM in otic patterning.

### *TCre-KO *embryos display defects in cell proliferation and survival

**Figure 3:**
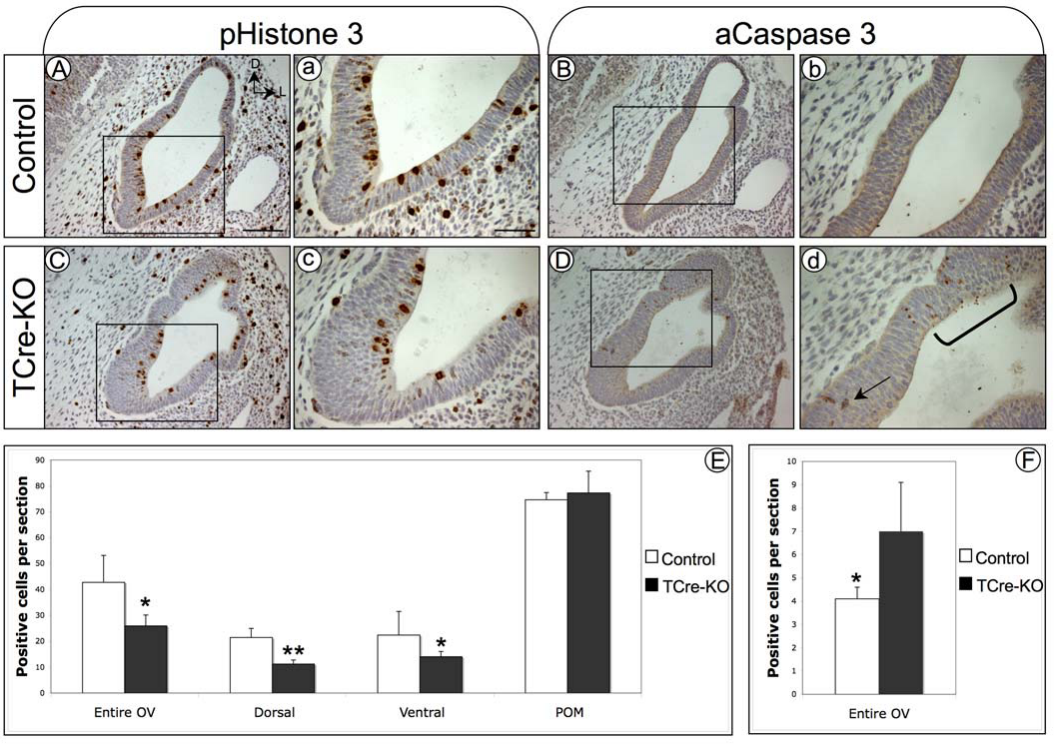
***TCre-KO *embryos exhibit defects in cell proliferation and survival**. Transverse sections at E11.5 of control (A) and *TCre-KO *(C) embryos labeled for mitotic cells using an Ab against phospho-Histone 3. *TCre-KO *embryos display a decrease in cell proliferation in the otic epithelium compared to controls upon quantification (E). Proliferation was reduced in both dorsal and ventral regions of the otic vesicle, but not in the POM. For the dorsal otic vesicle, 11.2 and 21.4 positive cells were observed for mutant and control, respectively, while 14.0 and 22.4 positive cells were observed observed for the ventral otic vesicle. *TCre-KO *embryos exhibit an increase in apoptosis at E11.5, identified by an Ab to active Caspase 3 (B, D). Arrow and bracketed area in (d) mark apoptotic cells. Quantification of apoptotic cells in control and mutant otic epithelium is shown (F). Asterisks indicate significant difference (* = *p *< 0.05, ** = *p *< 0.01, one-tailed, unpaired Student's t-test). Error bars represent 95% confidence interval. Boxed areas are magnified in a-d. Scale bar indicates 200 μm in (A), and 50 μm in (a).

*Tbx1 *has previously been shown to regulate the mitotic activity of the otic epithelium [24], indicating the abnormal inner ears seen in *TCre-KO *embryos may be due to defects in cell proliferation. To test this hypothesis, mutant and control embryos were analyzed at E11.5 using an antibody to the M-phase marker phospho-Histone 3 (Fig. 3A, C). This stage was chosen because it corresponds with the onset of morphological defects in the otic vesicle of *TCre-KO *embryos. Proliferating cells were counted in transverse sections through the entire otic vesicle of mutant and control embryos (n = 6). A significant reduction in proliferating cells was apparent (*p *= 0.011, Student's t-test), with an average of 25.9 and 42.7 positive cells per section in mutant versus control, respectively (Fig. 3E). Proliferating cells were also counted in the dorsal and ventral halves of the otic vesicle, and both of these regions displayed a decrease in proliferation in mutant compared to control embryos (Fig 3E, n = 6, *p *= 0.001 dorsal and *p *= 0.038 for ventral). Interestingly, no significant difference in proliferation was observed when dorsal and ventral halves of the *TCre-KO *mutants were compared directly (Fig. 3E). This suggests that cell proliferation is reduced broadly throughout the entire otic vesicle, as opposed to a localization of defects in the dorsal or ventral epithelium. We next tested whether proliferation of POM cells was affected in *TCre-KO *mutants. At E11.5, the cells of the POM have only just begun to condense around the otic vesicle and have not yet formed a well-defined population. We assessed cell proliferation in the POM of *TCre-KO *embryos by counting positive cells in an arbitrarily defined region of mesenchyme surrounding the otic vesicle. Proliferation was found to be unaffected in *TCre-KO *embryos compared to controls (n = 4, 77.3 and 74.6 positive cells per section in mutant and controls, respectively), suggesting that *Tbx1 *does not exert a tissue autonomous affect on proliferation of POM cells at this stage.

We next asked whether the otic epithelium of *TCre-KO *embryos displayed changes in cell survival using an antibody to the apoptotic marker activated Caspase 3. At E11.5, a significant increase in cell death was observed in the otic vesicle of *TCre-KO *embryos compared to controls (*p *= 0.035, Student's t-test, n = 4), with an average of 7.02 and 4.13 apoptotic cells per section in mutant versus control embryos, respectively (Fig. 3B, D, F). This indicates that *Tbx1 *expression in the POM has a modest affect on survival of the otic epithelium. Interestingly, apoptotic cells were not observed in the POM of *TCre-KO *or control embryos at this stage (Fig. 3B, D). These data suggest that *Tbx1 *has a role in regulating paracrine signaling to the otic epithelium prior to capsule formation.

### *Tbx1 *and *Brn4 *interact in the POM

**Figure 4:**
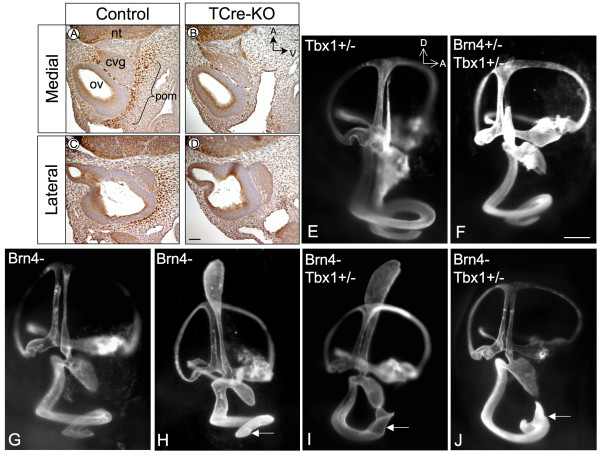
**Brn4 expression is reduced in the POM of *TCre-KO *embryos, and *Brn4*^-^;*Tbx1*^+/- ^embryo display defects in cochlea coiling**. Sagittal sections of control (A) and *TCre-KO *(B) embryos at E10.5 stained with an Ab against Brn4. Expression in the POM is lost in *TCre-KO *embryos, however neural tube (nt) expression is unaffected. *Brn4*^-^;*Tbx1*^+/- ^embryos display a shortened cochlear duct. Lateral views of paint-filled membranous labyrinths at E15.5–E16.0 of *Tbx1*^+/- ^(E), *Brn4*^+/-^;*Tbx1*^+/- ^(F), *Brn4*^-^(G, H), *Brn4*^-^;*Tbx1*^+/- ^(I, J). The fully developed mouse cochlea normally completes 1.75 turns, and all inner ears of *Tbx1*^+/- ^mutants and *Brn4*^+/-^;*Tbx1*^+/- ^exhibited normal cochlear growth (E, F). The majority of *Brn4*^- ^mutant inner ears also displayed a normal cochlea (G), while 20% displayed a slightly shortened cochlea (H). All inner ears *Brn4*^-^;*Tbx1*^+/- ^mutants exhibited a malformed cochlea that failed to form a second turn and instead displayed abnormal twisting or bifurcation (I, J). Arrows point to the cochlear duct in H-J. The endolymphatic sac in E, F, G and J was present but did not fill with paint. cvg, cochleovestibular ganglion; ov, otic vesicle; nt, neural tube; pom, periotic mesenchyme. Scale bar in D is 200 μm, scale bar in F is 350 μm.

A previous study showed that protein expression of Brn4, a marker for the POM, was lost in *Tbx1 *null mice but present in conditional mutants in which *Tbx1 *was ablated in the otic vesicle and pharyngeal endoderm [16]. This suggested that the POM domain of *Tbx1 *regulates *Brn4 *or proliferation of *Brn4*-expressing cells. To test this hypothesis, Brn4 expression was examined in *TCre-KO *embryos and was indeed reduced in the POM at E10.5 (Fig. 4A–D, n = 3).

We recently established a genetic interaction between *Tbx1 *and *Brn4 *in the inner ear, as adult *Brn4*^-^;*Tbx1*^+/- ^mice display a significant reduction in the number of cochlear turns compared to *Brn4*^- ^or *Tbx1*^+/- ^mice alone [19]. To determine the morphological basis of this interaction, the membranous labyrinth of compound mutants of *Tbx1 *and *Brn4 *was analyzed by paint-fill experiments. At E15.5, all *Tbx1*^+/- ^(n = 6) and *Brn4*^+/-^;*Tbx1*^+/- ^(n = 6) embryos exhibited grossly normal inner ear structures (Fig. 4E, F). Furthermore, the majority of *Brn4*^- ^mutants (*Brn4 *is X-linked) also exhibited normal inner ears with a completely coiled cochlea (8 out of 10 inner ears examined; Fig. 4G). Two *Brn4*^- ^mice displayed a slightly shortened cochlea of 1.5 turns (Fig. 4H). We then asked whether the addition of a single *Tbxl *null allele would affect the penetrance of the *Brn4 *null phenotype. All *Brn4*^-^;*Tbx1*^+/- ^mutants (n = 10) exhibited one cochlear turn followed by abnormal twisting and termination of the cochlear duct (Fig. 4I). In some cases, the cochlear duct bifurcated before terminating prematurely (Fig. 4J). These data indicate that *Tbx1 *may function in parallel with *Brn4 *to regulate a common downstream pathway important for cochlear growth.

### *Tbx1 *regulates RA metabolism in the POM

The defects seen in *TCre-KO *and *Brn4*^-^;*Tbx1*^+/- ^embryos suggest a non-autonomous role for *Tbx1 *and *Brn4 *in the POM during cochlear outgrowth. Because both genes are nuclear transcription factors, they may act on a common downstream target that signals to the otic vesicle. One likely candidate is retinoic acid (RA), which is known to be important for epithelial-mesenchymal interactions during inner ear development [20]. *Tbx1*^-/- ^embryos carrying a RA response element reporter construct [25] exhibited increased RA activity in the pharyngeal mesenchyme, including the POM, at E9.5 and E10.5 [26]. Further, expression of *Cyp26 *enzymes that catabolize active RA was reduced in these same tissues in *Tbx1*^-/- ^embryos [26,27]. We hypothesized that the inner ear defects present in the *TCre-KO *mutant might be due to abnormal RA metabolism caused by a reduction of *Cyp26 *gene expression. Of the three *Cyp26 *genes that catabolize RA, both *Cyp26a1 *and *Cyp26c1 *are co-expressed with *Tbx1 *in the POM [27]. To test if mesenchymal *Tbx1 *regulates these genes, expression analysis was performed on *TCre-KO *embryos.

**Figure 5:**
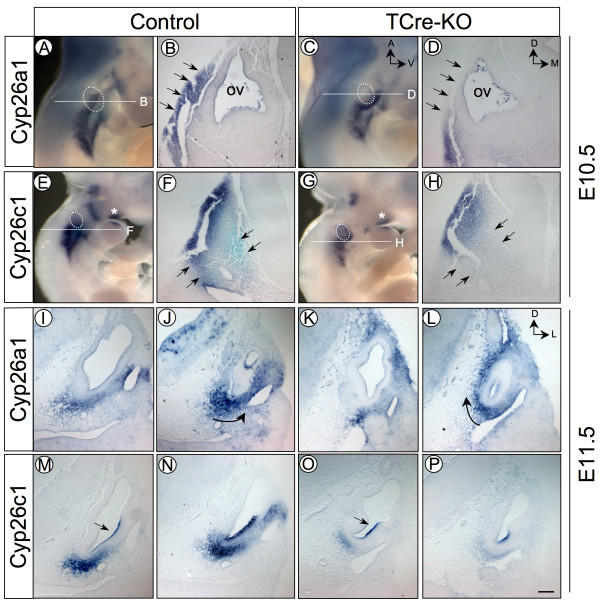
**Expression of *Cyp26 *genes is altered in *TCre-KO *embryos**. *Cyp26a1 *and *Cyp26c1 *expression detected by whole mount *in situ *hybridization at E10.5 in control and *TCre-KO *embryos (A-H). The plane of section in B, D, F and H is marked in A, C, E, and G, respectively. *Cyp26a1 *expression in the lateral POM (arrows in B) is absent in *TCre-KO *mutants (arrows in D), and appears shifted to a more ventral position (compare A an C). *Cyp26c1 *expression is strongly reduced in the mesenchyme of *TCre-KO *mutants (compare F and H), but 1^st ^arch expression is unaffected (asterisk in E and G). At E11.5, *Cyp26a1 *expression remains shifted medially in *TCre-KO *mutants compared to controls (I-L), especially in posterior sections through the growing cochlear duct (arrows in J and L). *Cyp26c1 *is strongly reduced in the ventral POM at this stage (M-P), but remains present in the otic epithelium (arrows in M and O). A dotted circle outlines the otic vesicle (ov) in A, C, E, G. Scale bar in P is 200 μm and applies to all panels except A, C, E, G.

*Cyp26a1 *and *Cyp26c1 *are expressed in similar patterns in the inner ear [28]. Both genes are restricted to the lateral and ventral POM at E10.5 and E11.5, which corresponds to the mesenchymal cells adjacent to the developing cochlear duct (Fig. 5B, F, I, J, M, N). *Cyp26c1 *is also found in the lateral wall of the cochlear duct epithelium (Fig. 5M, N). The expression of these genes in the POM significantly overlaps with *Tbx1 *expression at these stages (compare Additional file 2, Fig. S2E to Fig. 5A, E). At E10.5 in *TCre-KO *embryos, *Cyp26a1 *expression was missing from the lateral POM and appeared to be shifted to a more ventral position (Fig. 5A, C, n = 3). Transverse sections through the otic vesicle confirmed the absence of *Cyp26a1 *expression in the lateral POM (Fig. 5B, D). Further, expression of *Cyp26c1 *was strongly reduced in the mesenchyme at E10.5 in *TCre-KO *mutants, while first arch expression was unaffected (Fig. 5E, G, n = 3). The reduction of *Cyp26c1 *expression is particularly evident in the POM immediately posterior to the otic vesicle (Fig. 5F, H). Expression of *Cyp26b1*, which is predominately in the dorsal POM, was unchanged in *TCre-KO *mutants (data not shown).

The shift of *Cyp26a1 *expression from a lateral to a medial position was also apparent at E11.5 in *TCre-KO *embryos compared to controls (Fig. 5I–L, n = 3). This change in expression was more pronounced in posterior sections through the developing cochlear duct (Fig. 5J, L). Also at this stage, *Cyp26c1 *expression was strongly reduced in the ventral POM of *TCre-KO *mutants, while expression in the cochlear duct epithelium was unaffected (Fig. 5M–P, n = 3).

**Figure 6:**
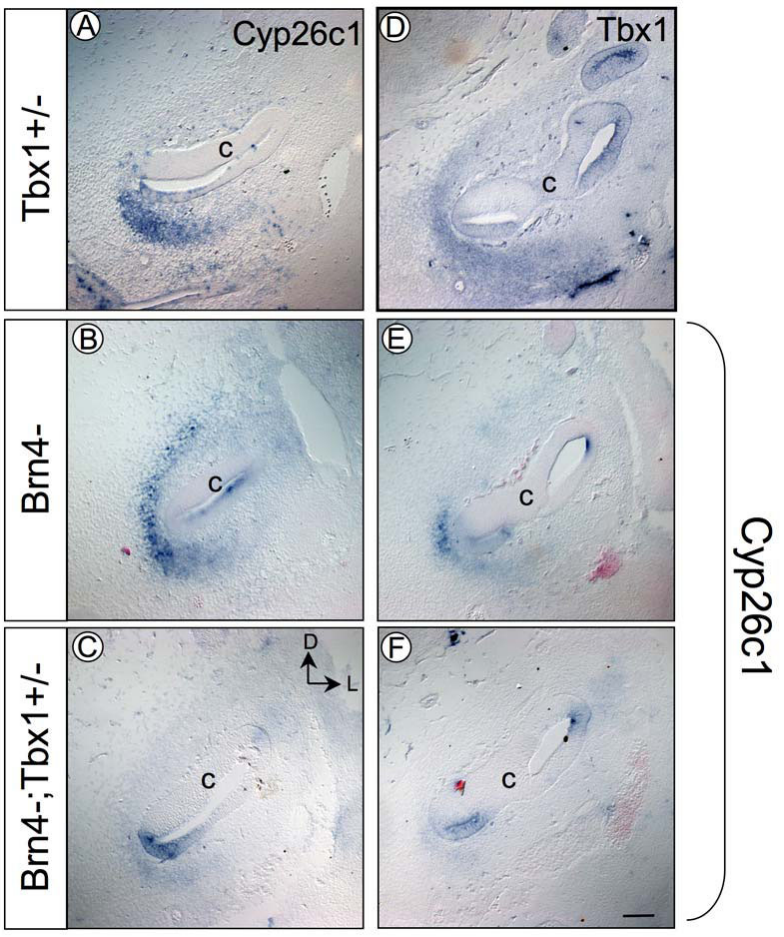
***Cyp26c1 *expression is reduced in *Brn4*^-^;*Tbx1*^+/- ^embryos**. Transverse sections through the cochlear duct (c) probed for *Cyp26c1 *(A-C, E-F) or *Tbx1 *(D) in *Tbx1*^+/-^, *Brn4*^- ^and *Brn4*^-^;*Tbx1*^+/- ^embryos at E12.5. *Cyp26c1 *expression is reduced in the ventromedial POM surrounding the cochlear duct of *Brn4*^-^;*Tbx1*^+/- ^embryos (C and F) compared to *Tbx1*^+/- ^(A) and *Brn4*^- ^(B and E) embryos. *Tbx1 *is expressed in this same region (D) and overlaps with *Cyp26c1 *expression. Expression of *Cyp26c1 *in the otic epithelium is unaffected in *Brn4*^-^;*Tbx1*^+/- ^embryos. Scale bar is 200 μm.

This data suggests that *Tbx1 *acts upstream of two *Cyp26 *genes expressed in the medial and ventral POM. To further test this hypothesis, expression of *Cyp26a1 *and *Cyp26c1 *was analyzed in *Tbx1*^+/-^, *Brn4*^- ^and *Brn4*^-^;*Tbx1*^+/- ^embryos. If *Brn4 *and *Tbx1 *both act upstream of a common target in the POM, then *Brn4*^-^;*Tbx1*^+/- ^embryos might display a similar change in *Cyp26a1 *and *Cyp26c1 *expression as seen in *TCre-KO *embryos. In *Tbx1*^+/-^, *Brn4*^- ^and *Brn4*^-^;*Tbx1*^+/- ^embryos at E12.5, expression of *Cyp26a1 *was unchanged (data not shown). However, *Cyp26c1 *expression was reduced in the ventral POM of *Brn4*^-^;*Tbx1*^+/- ^embryos compared to *Tbx1*^+/- ^and *Brn4*^- ^embryos (Fig. 6A–C, E–F). Further, *Cyp26c1 *is co-expressed with *Tbx1 *in the ventral POM surrounding the cochlear duct at this stage (Fig. 6A, D). Together with the results seen in *TCre-KO *mutants, these data indicate that *Cyp26c1 *is more sensitive to *Tbx1 *dosage than *Cyp26a1*. In addition, they suggest that *Tbx1 *and *Brn4 *act in concert to regulate RA activity in the POM, consistent with increased mesenchymal RA signaling observed in *Tbx1*^-/-^embryos.

## Discussion

### Distinct roles of *Tbx1 *in the otic vesicle and POM

During inner ear development, *Tbx1 *is expressed in the ventral mesenchyme cells that surround the otic vesicle and give rise to the cochlear capsule. *TCre-KO *embryos displayed malformations in both the otic capsule and membranous labyrinth, with ventral structures most severely affected. These results indicate that *Tbx1 *expression in the POM is essential for development of the epithelial structures of the inner ear. There are two potential explanations for this phenotype. It is possible that mesodermal *Tbx1 *is required for proper formation of the otic capsule, and abnormal condensation or differentiation of the POM prohibits proper outgrowth and coiling of the cochlea. An alternative, yet not mutually exclusive, explanation is that ablation of mesodermal *Tbx1 *disrupts signaling to the cochlear epithelium, altering the expression of genes critical to the formation of the membranous labyrinth and subsequently the otic capsule. This latter scenario is supported by the presence of physical malformations and a decrease in cell survival in the otic vesicle of *TCre-KO *embryos prior to capsule formation. We provide evidence that signaling to the otic epithelium occurs via RA, and propose that proper control of RA activity by the POM domain of *Tbx1 *is required for normal development of the inner ear.

The data from this and previous studies support distinct and independent roles for *Tbx1 *in the otic vesicle and POM, despite the close proximity of these tissues during development. Expression of *Tbx1 *in the otic vesicle functions just after induction of the otic placode, and is required for specification of neural versus sensory cell fate. This is accomplished via inhibition of neurogenic genes such as *Ngn1 *and *NeuroD*, while promoting expression of sensory markers such as *Otx1 *and *Bmp4 *[14,16]. In contrast, expression of *Tbx1 *in the POM does not appear to be required until after the major patterning events of the inner ear have occurred and the otic vesicle has acquired its axial identities. Indeed, sensory and neurogenic markers are expressed normally in the otic vesicle of *TCre-KO *embryos at E10.5. Due to the hypomorphic nature of the *TCre-KO *allele, we cannot exclude the possibility that sufficient *Tbx1 *expression exists at earlier stages to obscure a subtle patterning phenotype. However, lack of patterning defects in *Mesp1Cre-KO *using a different floxed allele [17,24] embryos argues against this alternative.

### Genes interacting with *Tbx1 *in the POM

We have previously identified a genetic interaction between *Tbx1 *and *Brn4 *in the POM [16,19]. Mice null for *Brn4 *display a reduction in the number of cochlear turns, although the penetrance of this phenotype is incomplete and is less severe than the cochlear defects observed in the *TCre-KO *mutant [12]. Brn4 protein expression was reduced in *TCre-KO *embryos at E10.5, indicating that *Tbx1 *may regulate transcription of *Brn4*. Alternatively, it is possible that Brn4 expression is lost due to a reduction in the number of POM cells in the *TCre-KO *mutant. Although no changes in proliferation or apoptosis were observed in the POM at E11.5, *Tbx1 *is expressed in only a subset of cells in the POM and confining the analysis to *Tbx1*-expressing mesenchymal cells may reveal a proliferation defect in this tissue. Nevertheless, the completely penetrant cochlear phenotype observed in *Brn4*^-^;*Tbx1*^+/- ^embryos together with the absence of detectable defects in *Brn4*^+/-^;*Tbx1*^+/- ^embryos suggest that these genes act in parallel pathways.

**Figure 7:**
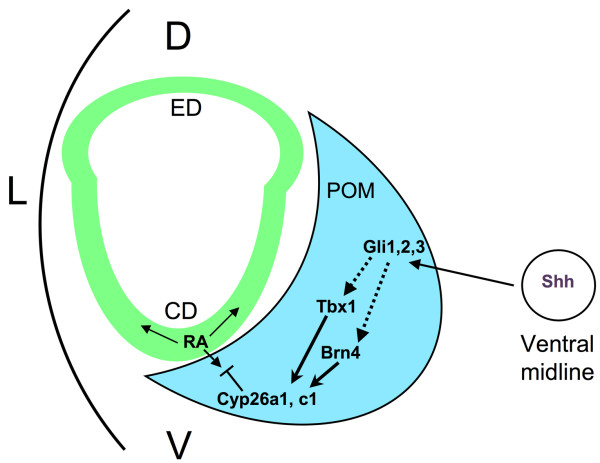
**Model of *Tbx1 *and *Brn4 *function in the POM**. Sonic hedgehog (Shh) secreted from the notochord (circle) and floor plate regulates both *Tbx1 *and *Brn4 *in the POM (blue). This signal is likely mediated by *Gli *genes expressed in the POM. *Tbx1 *and *Brn4 *activate members of the *Cyp26 *family also expressed in the POM. These genes catabolize active retinoic acid (RA) secreted from the otic vesicle (green), thereby regulating RA activity in the POM. *Brn4 *regulates expression of *Cyp26c1*, while *Tbx1 *regulates both *Cyp26a1 *and *Cyp26c1*. Strict control of RA dosage in the POM is likely crucial for proper cochlear coiling. CD, cochlear duct; ED, endolymphatic duct; POM, periotic mesenchyme.

As previously mentioned, Shh secreted from the ventral midline regulates expression of *Tbx1 *in the POM, and *Tbx1 *is expressed in the ventral POM closest to the source of Shh (the notochord and floor plate). We hypothesize that *Tbx1 *functions in parallel with *Brn4 *to modulate gene expression in a critical subset of POM cells on the ventral border of the otic vesicle, and that interactions between these cells and the otic epithelium are necessary for proper cochlea formation (Fig. 7). Recently, a model was proposed in which Shh functions in two phases to regulate cochlear duct morphogenesis [6]. In early development, Shh secreted from the ventral midline regulates patterning of the ventral otic vesicle by antagonizing the Gli3 repressor protein. In the later phase, Shh promotes outgrowth of the distal portion of the cochlear duct via recruitment of Gli activators. The cochlear defects seen in the mutants analyzed in this study indicate that *Tbx1 *and *Brn4 *likely act downstream of *Shh *in this second phase of development. Supporting this is the expression of mediators of *Shh *signaling, *Ptc1*, *Gli1*, *Gli2 *and *Gli3*, in the ventral POM [6]. In addition, the forkhead box transcription factors *Foxc1 *and *Foxc2 *are known to directly activate *Tbx1 *expression in the POM by binding to an enhancer, downstream of *Shh *[29]. This places these *Fox *genes downstream of *Gli *signaling but upstream of *Tbx1*.

### *Tbx1 *may regulate RA activity

Our data support a model in which *Brn4 *and *Tbx1 *act cooperatively on the same downstream target(s) to promote proper growth of the cochlear duct. Altered expression of *Cyp26 *genes in the POM of mutant embryos indicates that RA may be one of these targets (Fig. 7). In the ear, RA activity is regulated via metabolizing enzymes that are expressed in both the otic vesicle and the POM [28]. Expression of the *Raldh *genes is mainly confined to the epithelium, while the *Cyp26 *genes localize to the mesenchyme [20]. Expression of both *Cyp26a1 *and *Cyp26c1 *was altered in the POM at E10.5 and E11.5 in *TCre-KO *embryos, while *Cyp26c1 *was also reduced in *Brn4*^-^;*Tbx1*^+/- ^embryos at E12.5. These data indicate that *Tbx1*, and to a lesser extent *Brn4*, are required to maintain expression of *Cyp26a1 *and *Cyp26c1 *in the POM during inner ear development. Loss of *Tbx1 *in the POM also caused a decrease in cell proliferation and an increase in apoptosis in the otic epithelium at E11.5. These defects were not localized to the ventral epithelium, but were instead observed throughout the entire otic vesicle. While unexpected, this result may be due to the diffusible nature of RA, and provides an explanation for the reduction in size seen in dissected otic vesicles of *TCre-KO *embryos at this stage. Alternatively, it could be due to normal cell movement within the tissue.

Mouse mutants in which RA activity is increased during embryogenesis, either by null mutation of the *Cyp26 *genes or exposure to ectopic RA, display abnormal segmentation of the hindbrain [30-32]. Without the proper external cues emanating from this tissue, otic vesicle patterning and morphogenesis is abnormal [33]. Because of these early defects, however, the function of RA at later stages of ear development is not as well understood. Ectopic RA activity due to decreased catabolization could lead to the cochlear malformations seen in *TCre-KO *and *Brn4*^-^;*Tbx1*^+/- ^embryos via RA action on the otic vesicle, POM or both. Cyp26 enzymes expressed in the POM may function to inactivate excess RA produced by the epithelium, preventing abnormal regulation of RA target genes in the otic vesicle. Supporting this, adminstration of ectopic RA after otic specification is complete results in hypoplastic, malformed inner ears in both mouse and chick [34,35]. In addition, genes important for cochlear development were shown to be inhibited by increased RA levels [36,37]. It is also possible that genes in the POM are responsive to excess RA, leading to defects in remodeling around the growing cochlear duct and preventing proper cochlear coiling. This has been demonstrated by the inhibition of capsule chondrogenesis following RA treatment of cultured otic explants of mouse embryos [38].

## Conclusion

In summary, we have further described the genetic interaction between *Tbx1 *and *Brn4 *and shown that these genes regulate RA catabolizing genes in the POM during inner ear morphogenesis. Our results add to the evidence supporting a tissue non-autonomous role of the POM during development of the inner ear. Future studies will focus on gaining a better understanding of the relationship between *Tbx1 *and RA signaling in inner ear development, and identifying novel downstream targets of *Tbx1 *in the POM.

## Methods

### Experimental animals

The generation of the *Tbx1 *null allele and the *Tbx1*^*flox *^allele have been previously described [39]. *T-Cre *mice were obtained from Dr. Mark Lewandoski at the NIH [8] and crossed to *ROSA26 *reporter mice (Jackson) to analyze Cre activity. To generate *T-Cre;Tbx1*^*flox*/- ^embryos (*TCre-KO*), *T-Cre *transgenic mice were crossed to *Tbx1*^+/- ^mice on a mixed C57Bl6/Swiss Webster background to obtain *T-Cre;Tbx1*^+/- ^mice. These mice were crossed to *Tbx1*^*flox*/*flox *^mice on a mixed genetic background. *Tbx1*^+/- ^littermates were used as controls in all experiments. Mice were genotyped using primers to the *Cre *transgene (5' tcgatgcaacgagtgatgag 3' and 5' accaagtgacagcaatgctg 3') and the *Tbx1*^*flox *^allele (5' tcttcttggggctgtagact 3' and 5' tgactgtgctgaagtgcatc 3').

To generate *Brn4 *and *Tbx1 *compound mutants, *Brn4 *hemizygous males or heterozygous females in a mixed genetic background were crossed to *Tbx1*^+/- ^mice maintained on a C57BL/6 background [19]. These offspring were intercrossed to obtain the following genotypes: *Brn4*^+/-^, *Tbx1*^+/-^, *Brn4*^+/-^;*Tbx1*^+/-^, *Brn4*^-/- ^(or *Brn4*^-^), and *Brn4*^-/-^(*Brn4*^-^);*Tbx1*^+/-^.

### Immunohistochemistry

Embryos were isolated in cold PBS followed by fixation in 4% PFA overnight at 4°C, ethanol dehydration and embedding in paraffin wax. Seven μm thick sections were treated with either polyclonal rabbit Tbx1 antiserum (Zymed) 1:500, affinity-purified rabbit polyclonal anti-Brn4 antibodies [40]1:400, anti-cleaved Caspase 3 (R&D Systems) 1:1000, or anti-phospho-Histone 3 (Upstate) 1:200 in TBS/0.1% Triton X-100/5% goat serum/2%BSA. Sections were incubated for 1 h at room temperature and visualized with a biotinylated goat anti-rabbit IgG conjugate (1:200; DakoCytomation), avidin-biotin complex/HRP formation (DakoCytomation) and DAB/chromogen reaction (DakoCytomation). For histology, sections were stained with hematoxylin and eosin.

### RNA *in situ *hybridization

Analysis of *Tbx1 *expression was performed using a full-length mRNA probe cloned by PCR into pCRII-TOPO (Invitrogen). A probe specific to the region deleted in *Tbx1 *conditional mutants was also generated and exhibited similar results. The remaining probes were either obtained as previously described [16] or generated as approximately 1 kb PCR fragments amplified from E13.5 mouse cDNA. All forward primers contained T3 polymerase priming sequence and all reverse primers contained T7 polymerase priming sequence. PCR products were purified by the PCR Purification Kit (Qiagen). Anti-sense RNA was *in vitro *transcribed and labeled with the T7 RNA polymerase (Roche) and DIG RNA Labeling Mix (Roche) using the Digoxigenin labeling method. Labeled RNA probes were purified by LiCl_2 _precipitation and re-suspended in 20 μl of RNAse-free water (Gibco). *In situ *hybridization was performed whole mount or on sections using standard methods as previously described [41]. To generate histological sections following whole mount *in situ *hybridization, embryos were dehydrated through a graded ethanol series (70% EtOH for 5 minutes, 95% EtOH for 10 minutes, 2 times 100% EtOH for 5 minutes, 100% EtOH for 10 minutes, 2 times Xylene for 15 minutes), embedded in paraffin wax and sectioned at a thickness of 10 μm.

### Inner ear paint-fill

Embryos were isolated at E15.5 in PBS, cut below the forelimbs and placed in Bodian fixative (5 ml glacial acetic acid, 5 ml 37% formaldehyde, 15 ml H_2_0, 75 ml 100% EtOH) overnight at room temperature. Embryos were dehydrated in 100% ethanol overnight and cleared in methyl salicylate. Prior to injections, embryo heads were hemisected and the brain was removed. The inner ears were visualized by injecting 0.2% white Correction Fluid in methyl salicylate into the membranous labyrinth. The micropipette was inserted into the superior ampulla or the utricle depending on the ease of visualization of the lumen.

### Bone and cartilage staining

Skeletal staining of E17.5 embryos was performed as previously described [42]. Embryos were dissected and the skin removed followed by fixation in 100% EtOH overnight. Bone and cartilage were stained overnight with Alizarin red and Alcian blue, respectively. Loose tissue was removed by digestion in 2% KOH overnight, followed by destaining in 1% KOH/20% glycerol for 4–7 days. Embryos were transferred to 20% glycerol/20% EtOH overnight, followed by storage in 50% glycerol/50% EtOH.

## Authors' contributions

EMB designed and carried out the experiments, analyzed the results and drafted the manuscript. DCM carried out experiments, performed statistical analysis for the proliferation and apoptosis studies and aided with the *in situ *analysis of mutant embryos. VSA performed histological analysis of *TCre-KO *embryos and aided in analysis of the mutant phenotype. JSA generated the Tbx1^*flox *^allele needed for creation of the *TCre-KO *mutant. BEM conceived, funded and supervised the project, which was carried out in her laboratory. Both EMB and BEM critically read and revised the manuscript. All authors have read and approved the final manuscript.

## Supplementary Material

Additional file 1 **Figure S1. Conditional inactivation of *Tbx1 *in the mesoderm**. Cre activity identified by *LacZ *expression from *T-Cre *mice crossed to the *Rosa26 *reporter strain. At E9.0 (A) and E10.5 (B), Cre activity (blue cells) can be seen throughout the mesenchyme surrounding the otic vesicle but absent from the otic epithelium and cochleovestibular ganglion. *Tbx1 *expression in control (C, E) and *TCre-KO *(D, F) embryos at E9.5 and E10.5. Compared to controls, *Tbx1 *expression is strongly reduced throughout the mesenchyme in the *TCre-KO *at E9.5, however expression in both the otic vesicle and pharyngeal endoderm are unaffected (D). At E10.5, *Tbx1 *expression in the posterolateral otic vesicle is unchanged in both mutant and control embryos (E, F). *Tbx1 *expression in the POM is not evident in these sections as this domain is restricted to the ventromedial mesenchyme. cvg, cochleovestibular ganglion; ov, otic vesicle; pe, pharyngeal endoderm; pom, periotic mesenchyme. A-D are sagittal sections, E-F are coronal. Click here for file

Additional file 2 **Figure S2. *TCre-KO *and *Mesp1Cre-KO *embryos display similar inner ear phenotypes**. Paint filling of the membranous labyrinth of *TCre-KO *(A, B) and *Mesp1Cre-KO *(C, D) mutants. In both mutants, the cochlea abnormal and does not coil properly, while the vestibular system is sometimes hyoplastic. The most severely affected *TCre-KO *mutants (B) display only the hook region of the cochlea. *In situ *hybridization of for *Tbx1 *in *Mesp1Cre-KO *mutants (F) reveals reduced expression compared to control (E). However, similar to the *TCre-KO*, some *Tbx1 *expression in the mesenchyme remains. Click here for file
