# Supplementary figures and images for: Tbx1 and Brn4 regulate retinoic acid metabolic genes during cochlear morphogenesis
Source: BMC Dev Biol. 2009 May 29;9:31. doi: 10.1186/1471-213X-9-31 (PMC2700094; doi:10.1186/1471-213X-9-31)

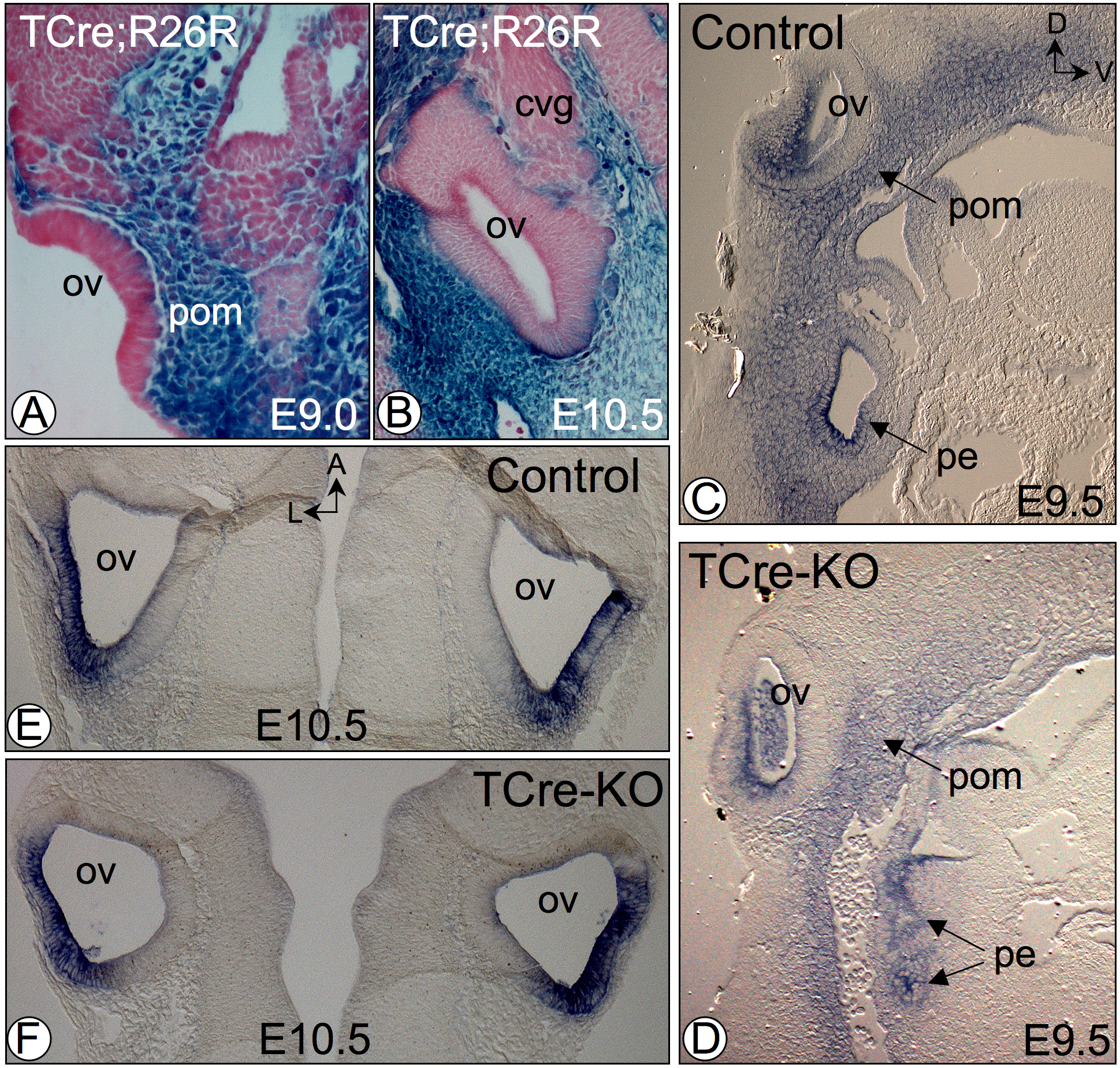

Supplement: Additional file 1 — Figure S1. Conditional inactivation of Tbx1 in the mesoderm. Cre activity identified by LacZ expression from T-Cre mice crossed to the Rosa26 reporter strain. At E9.0 (A) and E10.5 (B), Cre activity (blue cells) can be seen throughout the mesenchyme surrounding the otic vesicle but absent from the otic epithelium and cochleovestibular ganglion. Tbx1 expression in control (C, E) and TCre-KO (D, F) embryos at E9.5 and E10.5. Compared to controls, Tbx1 expression is strongly reduced throughout the mesenchyme in the TCre-KO at E9.5, however expression in both the otic vesicle and pharyngeal endoderm are unaffected (D). At E10.5, Tbx1 expression in the posterolateral otic vesicle is unchanged in both mutant and control embryos (E, F). Tbx1 expression in the POM is not evident in these sections as this domain is restricted to the ventromedial mesenchyme. cvg, cochleovestibular ganglion; ov, otic vesicle; pe, pharyngeal endoderm; pom, periotic mesenchyme. A-D are sagittal sections, E-F are coronal. [file 1471-213X-9-31-S1.png]

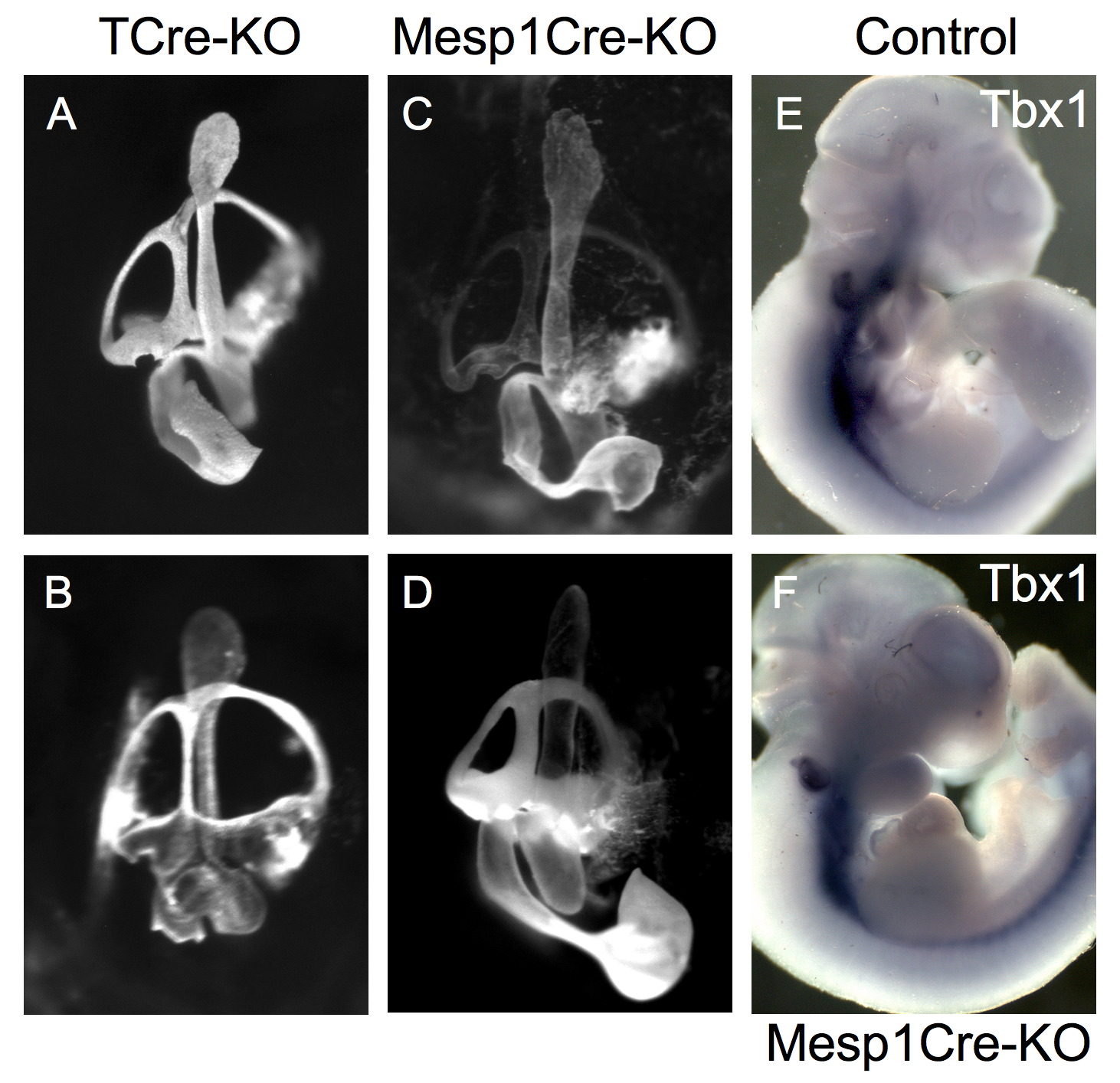

Supplement: Additional file 2 — Figure S2. TCre-KO and Mesp1Cre-KO embryos display similar inner ear phenotypes. Paint filling of the membranous labyrinth of TCre-KO (A, B) and Mesp1Cre-KO (C, D) mutants. In both mutants, the cochlea abnormal and does not coil properly, while the vestibular system is sometimes hyoplastic. The most severely affected TCre-KO mutants (B) display only the hook region of the cochlea. In situ hybridization of for Tbx1 in Mesp1Cre-KO mutants (F) reveals reduced expression compared to control (E). However, similar to the TCre-KO, some Tbx1 expression in the mesenchyme remains. [file 1471-213X-9-31-S2.png]
